# *Toxoplasma gondii* seroprevalence among pregnant women in Rabat, Morocco

**DOI:** 10.1186/s41182-021-00311-5

**Published:** 2021-03-08

**Authors:** Majda Laboudi, Zoubida Taghy, Oussama Duieb, François Peyron, Abderrahim Sadak

**Affiliations:** 1 Department of Parasitology, National Institute of Hygiene, 27 Avenue Ibn Batouta, BP: 769 Rabat, Agdal Morocco; 2 Faculty of Science, University Mohamed V, Rabat, Morocco; 3 Institut de Parasitologie et de Mycologie Médicale Hôpital de la Croix Rousse, Lyon, France

**Keywords:** Toxoplasmosis, *Toxoplasma gondii*, Pregnant women, Prevalence, Rabat, Morocco

## Abstract

**Background:**

Toxoplasmosis is an infectious disease caused by a protozoan parasite named *Toxoplasma gondii* (*T.gondii*). Pregnant women are considered one of the risk groups. The objective of this retrospective study is to provide an updated estimate of the seroprevalence of anti-*T. gondii* antibodies among a group of Moroccan pregnant women monitored at the Parasitology Laboratory of the National Institute of Hygiene in Rabat in Morocco.

**Methods:**

Serum samples were tested for the presence of specific anti-*T. gondii* immunoglobulin G (IgG) and immunoglobulin M (IgM) antibodies using indirect enzyme-linked immunosorbent assay (ELISA). Anti-*Toxoplasma* IgM- and IgG-positive cases were also evaluated with the anti-*Toxoplasma* IgG avidity test. All cases were evaluated according to the age, parity, and historical of abortion.

**Results:**

Among 677 pregnant women, 94.1% (637/677) were serologically screened for the first time and therefore had no knowledge of their serological status, and only 5.9% (40/677) were screened for the second or third time. The overall anti-*T. gondii* IgG and IgM seropositivity among the 637 pregnant women included in the study analysis was 43% (274/637) and 3.9% (25/637), respectively. The use of the IgG avidity test allowed excluding recent infection among 83% of cases with IgG and IgM positive sera. The mean age was 29.4 ± 6.3 years. The result of the bivariate analysis revealed that the age influenced significantly the seroprevalence rate, while the parity and the existence of previous spontaneous abortion did not have any significant statistical correlation with seropositivity to *T. gondii*.

**Conclusion:**

This study shows that 43% of pregnant women were positive and 57% of them had no antibody against the *T. gondii* infection. However, the pregnancy follow-up and the counseling of pregnant women remain essential for the prevention of congenital toxoplasmosis.

## Introduction

Toxoplasmosis is a parasitic disease caused by the protozoan *Toxoplasma gondii* (*T. gondii*) [1]. The Food and Agriculture Organization and the World Health Organization ranked toxoplasmosis fourth among the 24 most harmful food-borne pathogens [2]. In 80% of cases, the infection is asymptomatic in immunocompetent subjects [1]. However, the clinical aspects are serious in immunocompromised individuals and seronegative pregnant women (fetopathy) and newborns [3]. Human contamination occurs either by ingestion of oocysts (sporozoites) contained in water, vegetables, or fruits contaminated with cat excrement or by ingestion of cysts (bradyzoites) contained in raw or undercooked meat [4]. Other modes of transmission include transmission by blood transfusion, laboratory accidents, or organ transplantation. Vertical transmission, namely, maternal-fetal transmission, is likely to occur through tachyzoites in the maternal blood by transplacental passage and lead to congenital toxoplasmosis [5]. Toxoplasmosis is distributed worldwide in humans and warm-blooded animals [6]. One third of the human population is generally considered to be infected with *T. gondii*. The highest prevalence rates are found in the humid tropics [7, 8], and the lowest rates are found in the cold and Saharan regions [9]. Regional variations in seroprevalence are related to both climatic conditions, which may or may not favor the survival of oocysts, and factors such as the religious, cultural, and socioeconomic practices of each subpopulation [8].

The impact of toxoplasmosis on the health of the mother and the newborn should not be neglected. The surveillance, prevention, and control of toxoplasmosis are based mainly on research on IgG and IgM antibodies against *T. gondii.* The screening of the disease must be conducted early during pregnancy to allow the early detection of seroconversion that can lead to congenital toxoplasmosis. The clinical manifestations of congenital toxoplasmosis can be particularly severe when fetal contamination occurs during the first trimester of pregnancy, which is why it is recommended to perform toxoplasmosis serodiagnosis during the first months of pregnancy [10]. However, in the absence of a national toxoplasmosis surveillance program, it is common for a pregnant woman to arrive at the laboratory for toxoplasmic serology testing in the last trimester of pregnancy without knowledge of her previous serological status, even though the first and second trimesters are the most critical for fetal contamination.

In Morocco, a recent review reported there are only a few studies addressing the prevalence of toxoplasmosis [11]. Hence, we felt it was appropriate to update the data on the prevalence of toxoplasmosis among pregnant women in the region of Rabat and its surroundings and look for possible associations between seropositivity and the following factors: age, parity, and history of abortion.

## Materials and methods

### Study area

This descriptive retrospective study was conducted from April 2014 to September 2018 in Rabat that is located along the Atlantic Ocean, north-west of Morocco, and belonged at Rabat-Salé-Kénitra region (34° 02′ 00″ North, 6° 50′ 00″ West). Rabat is the capital of Morocco with an estimated population of 577,825 inhabitants (in 2014). The climate is Mediterranean with warm to hot dry summers and mild damp winters. Rabat belongs to the sub-humid bioclimatic zone with an average annual precipitation of 302.7 mm.

### Population study

This retrospective study was held on 677 asymptomatic pregnant women who were screened at the National Institute of Hygiene in Rabat, Morocco, for antenatal follow-up anti-*T. gondii.* Information about them such as age, parity, and history of abortion was reported from medical records. No personal identifiers were included in data collection forms.

### Inclusion/exclusion criteria

**Fig. 1:**
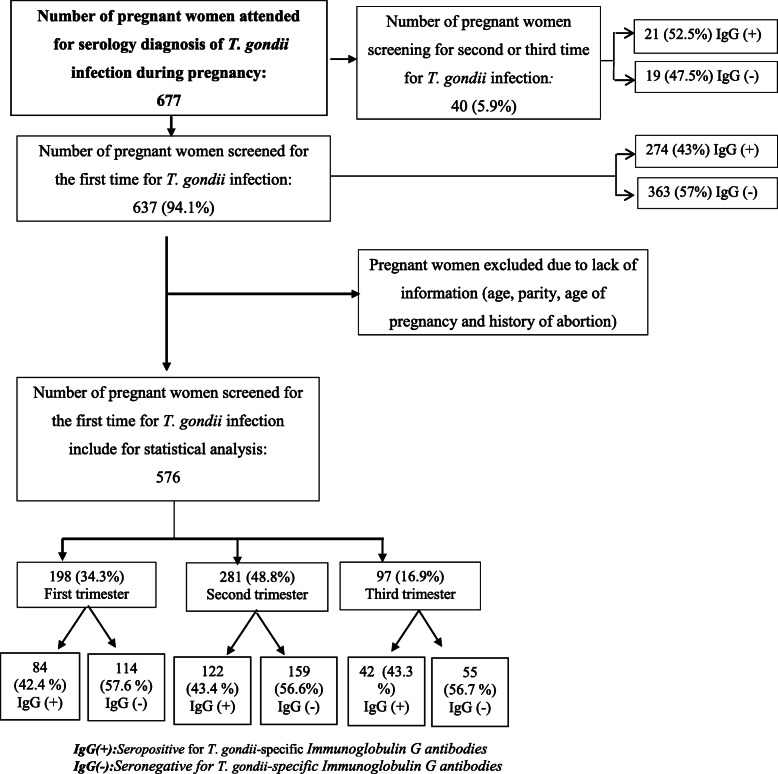
Flowchart explaining the selection of data for the analysis with details of their distribution of pregnant women by serological status for Immunoglobulin G *Toxoplasma gondii* infection antibodies

Among 677 pregnant women attended for serology diagnosis of *T. gondii* infection during pregnancy, only 576 who fulfilled the inclusion criteria were included in the analysis; All pregnant women who underwent pregnancy serological examination of toxoplasmosis for the first time during the studied time frame period were included. Additionally, the exclusion criteria were all pregnant women with uncompleted information (pregnant women without information of age, parity, and history of abortion) (Fig. 1).

### Serological evaluation

#### Conventional ELISA

The blood samples were collected from each pregnant woman and tube-coated EDTA and were afterwards centrifuged to remove plasma and harvested sera stored at − 20 °C until tested. Serological diagnosis was done following the ELISA technique (enzyme-linked immunosorbent assay) using “Platelia Toxo” kits IgG and IgM (Biorad, France) for immunoglobulin G (IgG) and immunoglobulin M(IgM) anti-toxoplasmic research according to the manufacturer’s protocol. Briefly, the sera were diluted serially and then distributed in the microplate wells which were coated by the *T. gondii* antigen. After incubation of 1 h at 37 °C, a monoclonal antibody labeled with peroxidase was added to the microplate wells as the conjugate. During the second incubation of 1 h at 37 °C, the labeled antibody binds to the serum IgG captured by the *T. gondii* antigen. The presence of immune-complexes (*T. gondii* antigen, IgG antibodies to *T. gondii*) and anti-IgG conjugate was demonstrated by the addition in each well of an enzymatic development solution. The optical density reading obtained with a spectrophotometer set at 450/620 nm is proportional to the amount of IgG antibodies to *T. gondii* present in the sample and is converted into IU/ml using a standard curve calibrated against. The positive cut-off value of IgG antibodies was defined at the upper limit of 9IU/ml. For the search of specific IgM antibodies, the immunocapture technique was performed. Anti-human μ-chain antibodies are coated on wells of the microplate. A mixture of the *T. gondii* antigen and the monoclonal anti-*T. gondii* antigen antibody labeled with peroxidase was used as the conjugate. The optical densities (OD) were obtained with a spectrophotometer set at 450/620 nm after stopping the reaction with a sulfuric acid solution. The cut-off value (CO) corresponds to the mean value of the OD of the cut-off control duplicates. The IgM results were calculated qualitatively, and it is expressed by the ratio of samples OD and CO. The sample was considered positive if the ratio was ≥ 1.

#### Avidity ELISA

All IgG- and IgM-positive samples included in the study were analyzed for IgG avidity at the first evaluation at a gestational age ≤ 20 weeks by the kit “Platelia Toxo avidity, BioRad,” according to the manufacturer’s protocol. The principle of this method relies on the measurement of the *avidity* of the IgG antibodies to *T. gondii*. The use of an agent as urea dissociating the link antigen/antibody in parallel with the usual technique of IgG antibodies measurement allows comparison of the optical density (OD) obtained after dissociating agent action and OD obtained without dissociating agent action. The avidity is considered low when the antigen/antibody link is easily dissociated. The optical density reading obtained with a spectrophotometer set at 450/620 nm. The diagnostic value was defined as avidity index (AI). The avidity index was determined as the following criteria: A low index of avidity below 0.4 does not exclude a recent primary infection of less than 20 weeks while high index avidity or equal to 0.5 can be excluded. In case of intermediate index of avidity (0.4 ≤ AI < 0.5), an assay on a second sample was recommended.

### Statistical analysis

The data input was done on Microsoft Office Excel 2010, and the analysis was performed using EpiInfo (ver. 2007 CDC, USA) Software. A descriptive analysis of the data was made to identify the characteristics of the different variables studied in EpiInfo. The chi-square test (bivariate test) from EpiInfo was used to determine the associated seroprevalence of *T. gondii*. Statistical significance was set to a value of *p* < 0.05.

## Results

### Demographic characteristics of pregnant women

In this retrospective study, among 637 pregnant women aged between 16 and 46 years were screened for the first time for *T. gondii infection*, only 576 were included in analysis, and they have complete information about age, parity, and history of abortion. The average age was 29.4 ± 6.3 years. The most of pregnant women were in the age groups 16–25 years and 26–30 years. We note that the majority of women screened were nulliparous 47.8% (275/576) followed by primiparous 45.1% (260/576) and multiparous women 41(7.1%). About 34.3% (198/576) of the pregnant women were in their first trimester, and 48.8% (281/576) and 16.9% (97/576) in the second and third trimesters, respectively (Table 1).

**Table 1:** Sociodemographic characteristics of pregnant women

Characteristic	Numbers	(%)	95% CI*
**Age ( years) mean: 29.4 ± 6.3**			
16–25	189	32.8	29.0–36.8
26–30	144	25.0	21.6–28.8
31–35	138	24.0	20.6–27.7
36–46	105	18.2	15.2–21.7
**Age of pregnancy**			
First trimester	198	31.1	27.5–34.9
Second trimester	281	44.1	40.2–48.1
Third trimester	97	15.2	12.6–18.3
**Parity**			
Nulliparous	275	47.8	47.7–51.9
Primiparous (1 P**)	260	45.1	92.9–29.7
Multiparous (≥ 2P**	41	7.1	100.0–30.2
**History of abortion**			
No abortion	446	77.4	73.8–80.7
One abortion	95	16.5	13.6–19.8
Two or more abortion	35	6.1	4.3–8.4

*CI* confidence interval  ** : Number of pregnancy .≥ 2P**: equal or more than  two pregnancy

### Serological status of pregnant women

According to the serological status for *T. gondii* infection, among 677 pregnant women, 94.1% (637 /677) [95%CI 92.0–95.7%] were serologically screened for the first time and therefore ignored their serological status, and 5.9% (40/677) [95%CI 4.0–9.0%] were screened for the second or third time (Fig. 1).

The results showed that the *T. gondii* seroprevalence of specific IgG was 43% (274/637) [95% CI 39.1–47.0%] while 57% (363/637) [95% CI 53.0–60.9%] were seronegative for anti-*T. gondii*-specific IgG antibodies. Furthermore, the percentage of *T. gondii* IgM-positive antibodies were 3.9% (25/637) [3.0–6.3%].

Among 25 pregnant women who had a positive IgG and IgM *T. gondii* infection during pregnancy, 18 cases are in their first month of pregnancy and are submitted to the avidity test***.*** The results of the avidity test are shown in Table 2. Anti-*T. gondii* IgG avidity in the majority of patients studied 83.3% (15/18) had high avidity while a low avidity was detected in two patients (Table 2).

**Table 2:** Comparison of results of IgM ELISA with IgG avidity test in 18 serum samples taken from pregnant women in Rabat region in Morocco

	IgG Avidity ELISA (%)		
	Low	Borderline	Higher
**IgG and IgM positive**	2 (11.1%)	1 (5.6%)	15 (83.3%)

### Risk factors associated with *T. gondii* infection

**Fig. 2:**
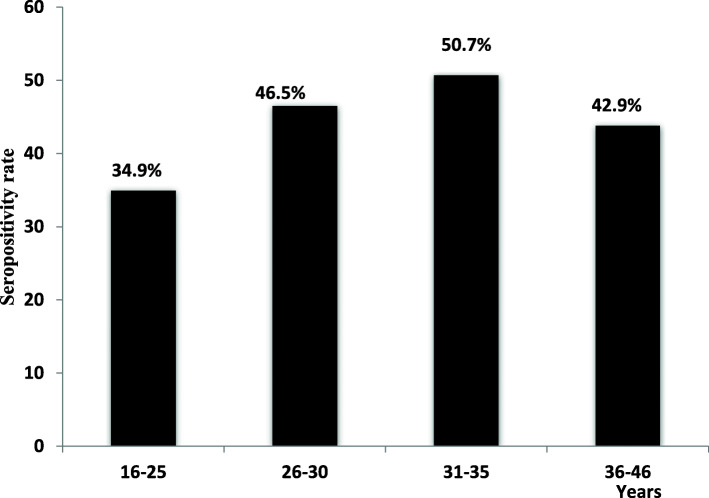
Distribution of pregnant women by serostatus for IgG *T. gondii* antibodies in various age group (years)

Seroprevalence of the parasite in the pregnant women based on various age groups, parity, and history of abortion is shown in Table 3. The results of bivariate analysis of age associated with *seropositivity* showed a significant difference in both the parasitic infection was more prevalent in the age group 31–35 years (50.7%) (70/138) followed by age group 26–30 years 46.5% (67/144) and 36–46 years 42.9% (56/105). The lowest percentage of *T. gondii* infection is found for women in the age group 16–25 years with about 35.7% (Fig. 2). This result is statically significant (*p value* = 0.0276)*.*

**Table 3:** Seroprevalence of toxoplasmosis according to age, parity, and history of abortion among pregnant women in Rabat region

Variable	***Toxoplasma*** seroprevalence		***X***^**2**^	***p*** value
	Seropositivity, ***n*** (%)	Seronegativity, ***n*** (%)		
**Age**				
16–25	66 (34.9%)	123 (65.1%)	9.1283	0.0276*
26–30	67 (46.5%)	77 (53.5%)		
31–35	70 (50.7%)	68 (49.3%)		
36–46	45 (42.9%)	60 (57.1%)		
Age of pregnancy				
First trimester	84 (42.4%)	114 (57.6%)	.0495	0.9756
Second trimester	122 (43.4%)	159 (56.6%)		
Third trimester	42 (43.3%)	55 (56.7%)		
**Paritiy**				
Nulliparous	110 (40.0%)	165 (60.0%)	2.5627	0.2777
Primparous (1 P^a^)	117 (45.0%)	143 (55.0%)		
Multiparous (≥ 2P^a^	21 (51.2%)	20 (48.8%)		
No abortion	188 (42.2%)	258 (57.8%)	.7715	0.6799
**History of abortion**				
One abortion	43 (45.3%)	52 (54.7%)		
Two or more abortion	17 (48.6%)	18 (51.4%)		

*NS statistically significant, ^a^Number of pregnancy

Multiparous women are the most immune for *T. gondii* infection with 51.2% (21/41), followed by primiparous 45% (117/260) and nulliparous 40% (110/275) (*p* value = 0.2777).

Regarding the history of abortion, among women who have had more than one abortion, 48.6% (17/35) are found to be positive for *T. gondii* infection compared to the women who had no abortion (42.2%) (188/446). Accordingly, even though a total IgG seropositivity was increased in pregnant women with a history of abortion, there are no significant associations observed between IgG seropositivity and the number of abortions (*p* value = 0.6799). On the other hand, a total IgG seropositivity rate was not more varied with the age of pregnancy (Table 3).

## Discussion

In Morocco, there are very few documented data on *T. gondii* seroprevalence [11]. Adequate information on the prevalence and transmission routes would provide proper risk assessment options for pregnant women and would be helpful in the planning and implementation of control and preventive strategies for toxoplasmosis disease.

The *T. gondii* seroprevalence among pregnant women in the study area was 43%. Comparing the levels of seropositive IgG antibodies obtained in our study with those in previous studies conducted in 2007 and 2014, which were 51% and 47%, respectively, in the same region studied [11], we noticed a decrease in the seroprevalence during the last 5 years. A previous study showed that the majority of pregnant women with anti-toxoplasma antibodies have continuous contact with the soil in activities such as gardening, agricultural activities, or other activities [12]. Therefore, the noticeable decline in the seroprevalence may be due to the regression of contact with the soil. In addition, changes in lifestyle that are related to development, hygiene measures, and higher levels of education can contribute to reducing this prevalence.

When we compared our findings to those of previous studies reported in different cities in Morocco, we found that *T. gondii* seroprevalence in these regions continues to be higher than that in cities at lower altitudes and with higher humidity in Morocco, such as Fes, where the prevalence is 39.7% [13]. It is also higher than those reported in Nador, Tetouan, and Kenitra, where the seroprevalences are 34.3%, 42%, and 37.7%, respectively. However, the current finding indicates that the seroprevalence remains lower than that reported in Essaouira (48%) [14]. In addition, our results remain close to those reported in the two neighboring Maghreb countries: Tunisia (45.6%) [15] and Algeria (47.8%) [16]. This could be explained by the fact that Morocco, Tunisia, and Algeria share the same culinary habits, cultural habits, and climatic conditions. However, the results of other surveys show a lower and higher prevalence in different areas in Africa, in Europe, and in Asia: 27% in Sudan [17], 35.6% in Ethiopia [18], 44% in Tanzania [19], 47% in Benin [20], 13.8% in Italy [21], 31.5% in Austria [22], 55.8% in Romania [23], 31% in Turkey [24], 33% in Iran [25], 34.5% in Pakistan [26], 82.6% in Lebanon [27], and 35.8% in Peru [28]. This variation in the rate of *T. gondii* infection between countries and regions could be attributed to dietary habits, health standards, lack of awareness of disease transmission, and the socioeconomic level. Improvements in hygiene conditions and farming systems, together with increased socioeconomic levels, have led to a declining seroprevalence in most industrialized countries [10].

On the other hand, our findings indicate that 57% are seronegative and are therefore likely to be infected during pregnancy, so serological monitoring is required during each trimester with prophylaxis to follow throughout the pregnancy. However, we noted that only 5.9% of all pregnant women were undergoing *T. gondii* antibody screening, with 47.5% seronegativity, and among the seronegative cases, 15.2% were in their third trimester when they underwent screening for *T. gondii* antibodies for the first time. Hence, in Morocco, no systematic prenatal toxoplasmosis screening has been set up. The health system does not have a monitoring program for toxoplasmosis. Therefore, there is no follow-up of *T. gondii* seronegative pregnant women to properly control the risk of infection with congenital toxoplasmosis until delivery. Moreover, serological screening for *T. gondii* infection in our country is still considered a biological test not required by doctors. Thus, the serological diagnosis of *T. gondii* infection is not always requested at the first prenatal visit, and the follow-up of pregnant women is poorly conducted or is not conducted at all. Besides, the lack of knowledge of toxoplasmosis among pregnant women has been reported previously as a main risk factor for contracting disease. Only 5.9% were aware of toxoplasmosis. The lack of knowledge may put unaware women to not follow-up the serology and put them in a high-risk group, susceptible to contract *T. gondii* infection during pregnancy leading to an acute infection [12]. On the other hand, a recent study reported a moderate knowledge of toxoplasmosis among health professional including physicians and nurses on this parasitic infection and thus failed to provide sufficient information to the pregnant women [29]. Hence, healthcare professionals should advise patients to follow-up the serology specially for the seronegative pregnant women in order to avoid seroconversion during pregnancy*.*

Indeed, serological screening is considered the key element in the prevention of congenital toxoplasmosis. In fact, monthly serology for seronegative pregnancy is mandatory until delivery. The interpretation of the serological results must be carried out by a competent and experienced biologist, since from the conclusions of the serological analysis, all subsequent prenatal and postnatal behavior is ensured [30]. Even when primary infection occurs during pregnancy, early diagnosis and treatment can reduce the frequency and severity of the disease in neonates [31].

The current study reveals that among seropositive women, the majority were found to have a chronic or past infection based on serology of IgM antibodies. However, 18 (2.8%) of the 637 pregnant women consulting in their first trimester were susceptible to a recent infection, and only two (2) subjects were positive for IgM with a low avidity using the avidity IgG method. This test excluded 83.3% of pregnant women. Therefore, IgM positivity does not always predict an acute infection. IgG avidity tests in addition to IgM and IgG antibody testing should be performed in the first trimester of pregnancy. The avidity test seems necessary to confirm or exclude an infection of less than 4 months in pregnancy, which is likely to lead to congenital toxoplasmosis [32, 33].

In this study, the risk of contracting *T. gondii* increased with the age of the pregnant woman; increased odds of having *T. gondii* infection were observed in the groups of pregnant women aged ≥ 25 years compared to the group of pregnant women aged 16–25 years. This finding is consistent with the studies conducted in Fes [13] and in Rabat [34], where the prevalence did not increase linearly with age. In contrast, our results are not in agreement with those found in France [35], where a positive correlation between seroprevalence and age was found; our results are probably due to the low number of women in the sample of our study.

## Conclusion

This descriptive study provides key and update baseline data about the *T. gondii* seroprevalence among pregnant women in the region of Rabat, Morocco. In this study, it is noted that the number of non-immunized pregnant women increases during the last years in the region studied. Therefore, the risk of fetal infection has increased because the possibility of seroconversion during pregnancy is higher among pregnant women. On the other hand, we report that few pregnant women have followed-up of toxoplasmosis during pregnancy especially among the number of non-immunized pregnant. Undoubtedly, a national program of screening and surveillance of seronegative pregnant women is required.

### Limitations

Our study presents some limitations mainly due to the fact that these data are derived from a retrospective evaluation of pregnant women. For this reason, important data, such as possible risk factors for *T. gondii* infection (consumption of uncooked meat, contact with cats, or other animals…) are not available. Furthermore, some factors could explain the regression of *T. gondii* IgG exposure; however, these factors were not ascertained in this study and constitute one of the limitation of the survey. Hence, there is a need for a national sample survey estimating the real potential burden of this infection on maternal and its impact on fetal health. Despite these limitations, we have for the first time been able to report the number of pregnant women who follow-up the screening of toxoplasmosis.

## Data Availability

The datasets used during the current study are available from the corresponding author on reasonable request.

## Abbreviations

AI: Avidity index
CO: Cut-off value
ELISA: Enzyme-linked immunosorbent assay
EDTA: Ethylenediaminetetra-acetic acid
IgG: Immunoglobulin G
IgM: Immunoglobulin M
OD: Optical density
*T. gondii*: *Toxoplasma gondii*
